# The RETA Benchmark for Retinal Vascular Tree Analysis

**DOI:** 10.1038/s41597-022-01507-y

**Published:** 2022-07-11

**Authors:** Xingzheng Lyu, Li Cheng, Sanyuan Zhang

**Affiliations:** 1 College of Computer Science and Technology, Zhejiang University, Hangzhou, 310027 China; 2 Department of Electrical and Computer Engineering, University of Alberta, Edmonton, T6G 1H9 Canada

**Keywords:** Medical imaging, Eye abnormalities, Machine learning, Eye manifestations

## Abstract

Topological and geometrical analysis of retinal blood vessels could be a cost-effective way to detect various common diseases. Automated vessel segmentation and vascular tree analysis models require powerful generalization capability in clinical applications. In this work, we constructed a novel benchmark RETA with 81 labelled vessel masks aiming to facilitate retinal vessel analysis. A semi-automated coarse-to-fine workflow was proposed for vessel annotation task. During database construction, we strived to control inter-annotator and intra-annotator variability by means of multi-stage annotation and label disambiguation on self-developed dedicated software. In addition to binary vessel masks, we obtained other types of annotations including artery/vein masks, vascular skeletons, bifurcations, trees and abnormalities. Subjective and objective quality validations of the annotated vessel masks demonstrated significantly improved quality over the existing open datasets. Our annotation software is also made publicly available serving the purpose of pixel-level vessel visualization. Researchers could develop vessel segmentation algorithms and evaluate segmentation performance using RETA. Moreover, it might promote the study of cross-modality tubular structure segmentation and analysis.

## Abstract (summary)

**Table:** 

Measurement(s)	Retina blood vessel • Abnormal Retinal vascular morphology • Retinal vascular tree
Technology Type(s)	Image Segmentation • Digital Image Analysis • Supervised Machine Learning • Computer Application • Computer-Aided Diagnosis • Image Processing • Graph-based Analysis • Neural Network

## Background & Summary

Vessel calibre is one of retinal vascular biomarkers for early detection of microvascular and macrovascular diseases, such as diabetic retinopathy (DR)^1^, hypertensive retinopathy and stroke^2,3^. Colour fundus image, as a two-dimensional (2-D) colour photograph captured by fundus camera, is a non-invasive and widely-used ophthalmic imaging^4^ to evaluate retinal vascular abnormalities (*e.g*. calibre change, tortuosity alteration^5^, neovascularization and arteriovenous nicking (AVN)^6^). For decades, researchers have been utilising computer-aided methods to automatically segment retinal blood vessels including arterioles and venules from retinal images^7–9^. The state-of-the-art deep learning (DL) method demonstrates superior capability to classify each pixel of a fundus image into binary (vessel/background) or multiple (artery/vein/background) classes^9^. Traditional vascular analysis procedure makes use of the segmented vessels and focuses on analysing vascular structures and measuring vascular biomarkers. It usually consists of several image processing modules^10^ including vessel skeletonization, vascular anatomical landmark (bifurcations and crossovers) identification, arterial/venous (A/V) vascular tree tracking, vessel diameter measurement and arteriolar-to-venular diameter ratio^11^ calculation. Compared with a recent DL-based vascular analysis model^3^, medical diagnostics from the segmentation-based approach are technically interpretable and more acceptable by professionals. However, cross-dataset evaluation results indicate generalization ability of present segmentation model is still the bottleneck^12^ due to domain gap between source dataset and target dataset^13,14^. Manual error correction seems inevitable so far if conducting any follow-up analysis of the segmentation-based vascular analysis programme.

Training set associated factors that affect model robustness are the scale of training set and the amount of noisy labels. To the best of our knowledge, there are more than ten open-access datasets for binary vessel segmentation. Some of them kindly provide A/V labels. There are also some image sets developed for vascular network analysis concerning vascular keypoint/junction detection^15,16^ and vascular tree tracking^17,18^. In vascular trees, each tree starts from a starting vertex near optic disk (OD) and terminates at an ending vertex. Vascular keypoints are selected as the graph vertices. Vascular network analysis^10,17,18^ is an effective technique for A/V tree separation and branching complexity analysis. Based on these facts, open vessel datasets are considerably valuable for developing automated algorithms but far from enough. Alternatively, image augmentation techniques, for example image rotation and synthesis^19^, could increase the number of training samples. But the frequently argued issue of annotation noise^20,21^ is unsolved because of the inter-annotator and intra-annotator variabilities. Pixel-level vessel annotation unfortunately is quite expensive and time-consuming. Only well-trained experts who use specialized vessel annotation software, like VAMPIRE annotation tool^22^, equipped with user-friendly labelling tools could guarantee the quality of vessel annotations.

**Fig. 1:**
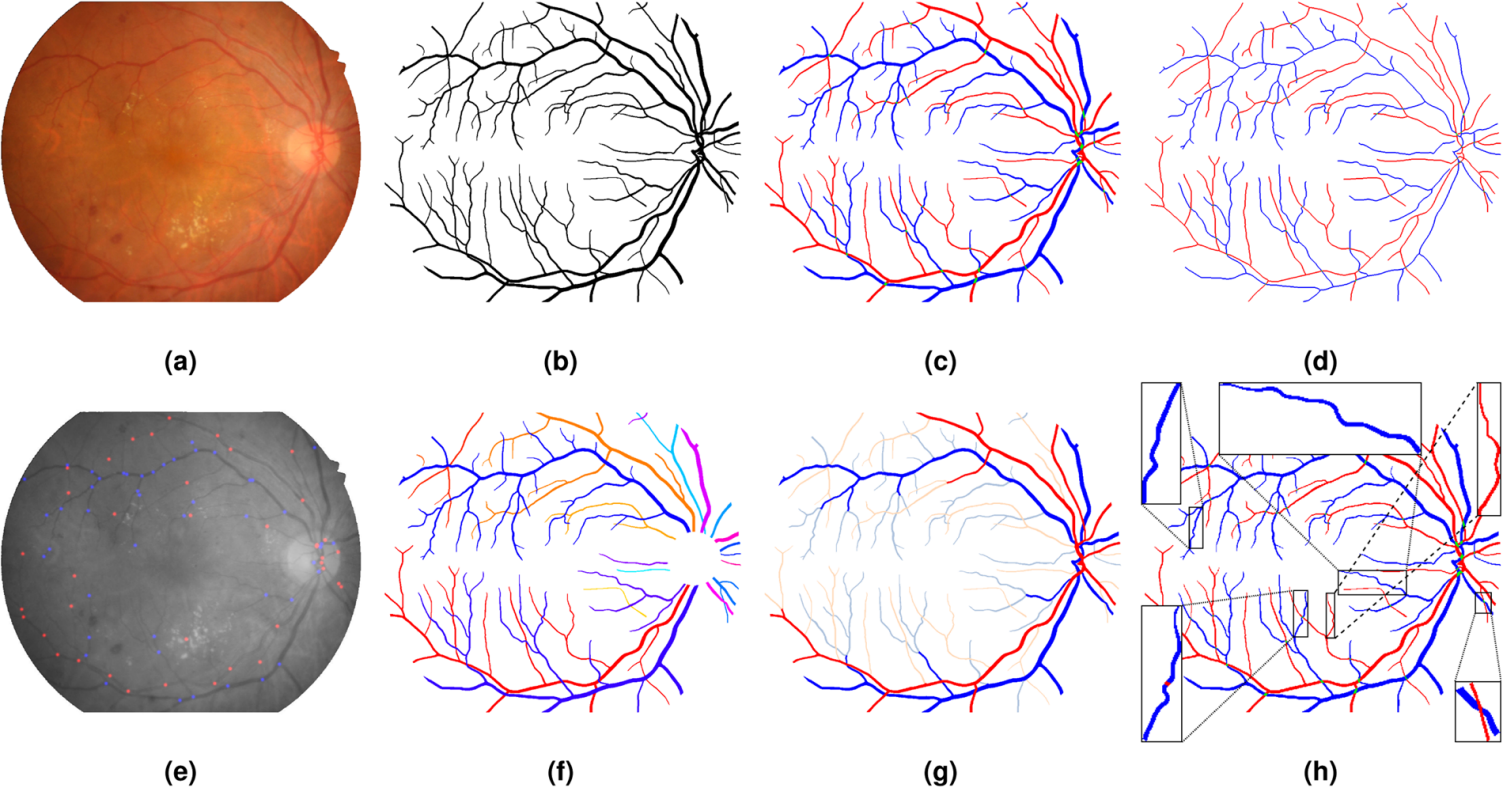
An overview of vessel annotations in RETA Benchmark. (**a**) Colour fundus image without black image background. (**b**) Binary blood vessel mask. (**c**) A/V vessel mask (red : arterial vessel pixels; blue : venous vessel pixels; green : overlapping pixels of the arteries and veins). (**d**) Vessel skeletons (vessel centreline image is morphologically dilated with 1-pixel disk-shaped structuring element for a better visualization). (**e**) Vascular bifurcations superimposed on the grey-scale fundus image (red and blue points represent arterial and venous bifurcations respectively). (**f**) Vascular trees with the OD region removed (each geometric tree is encoded by a specific colour). (**g**) Thick (blue & red) and thin (shades of blue & red) vessels distinguished by the vascular calibre. (**h**) Vascular abnormalities highlighted on the A/V mask. Vascular disease label of the bottom-right bounding box is more likely to be AVN and the remaining bounding boxes probably highlight vascular tortuosity.

In this work, we contributed a novel benchmark RETA for **RE**tinal vascular **T**ree **A**nalysis following the guideline of benchmark construction protocols^23^. RETA contains 81 images derived from the first subset of IDRiD dataset^24,25^ and corresponding pixel-level blood vessel masks. A self-developed MATLAB-based interactive tool named as Computer Aided Retinal Labelling (CARL) is used for vessel annotation, validation and visualization. The designed vascular annotation pipeline involves pixel-level, structure-level and network-level stages. Figure 1(a,b) show a fundus image and labelled vessel mask. The intermediate outputs from different annotation stages are shown in Fig. 1(c,h). The potential reuse value for our RETA benchmark includes.

- **Develop and evaluate retinal blood vessel segmentation approaches**. The presence of different kinds of diabetic lesions like microaneurysms, soft exudates, hard exudates and hemorrhages in colour fundus images makes the vessel segmentation a more challenging task.
- **Build multi-task automated retinal image analysis models**. Pixel-level annotations of the OD and diabetic lesions are already available in the IDRiD dataset. Developing a multi-task DL model to learn geographic relationship between the retinal vessels and other objects could be an interesting research topic.
- **Segment similar tubular structures in other imaging modalities**. Adapt feature domain from colour fundus photography to other medical imaging modalities, like optical coherence tomography angiography and magnetic resonance angiography, and segment tubular structures using a domain adaptation or transfer learning approach^14,26^.
- **Decouple and analyse retinal vascular trees**. Track the A/V trees to obtain a correct topology estimation. Measure topological and geometrical features of traced vascular trees^27^, such as branchpoint density, vascular calibre, fractals and tortuosity.

## Methods

**Fig. 2:**
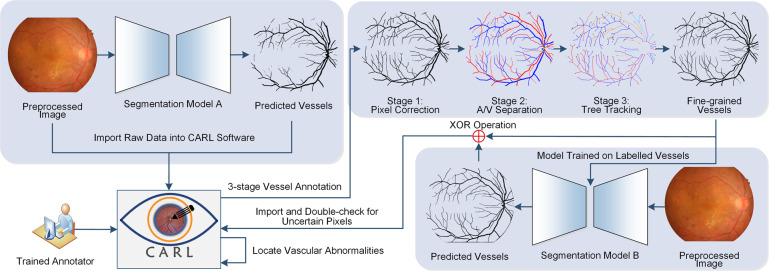
The proposed workflow for generating fine-grained vessel annotations. Segmentation models A and B are two automated binary vessel segmentation models used to predict vessel pixels from the preprocessed retinal images. Stage 1 is pixel-level manual annotation on raw vessel predictions from model A. Stage 2 is structure-level A/V segment identification. In Stage 3, we validated A/V annotations from Stage 2 by means of network-level analysis. Specifically, we tracked every single tree starting from the OD boundary. Different trees are encoded in unique colours. Anatomical landmark points of each tree are highlighted, among which red dots present starting points. We identified and double-checked ambiguous pixel labels through mapping vessel predictions from the model B to the original manual annotations. We also scanned annotated vessel structures to detect vascular abnormalities where noisy pixels may hide.

A semi-automated method is designed to depict the retinal blood vessels from coarse to fine in the fundus image. Figure 2 displays an overview of the proposed workflow. Two DL-based semantic segmentation models are used to automatically generate vessel segmentation images. The CARL software is used for interactive vessel annotation and validation. In particular, the top right block of Fig. 2 is a 3-stage vessel labelling strategy to assure precise locations of vessel pixels within the fundus image. The first stage is pixel-level annotation. One human annotator worked extensive manual vessel correction over computer predicted image from segmentation model A. Then, structure-level segment labelling was performed to classify all vessel segments into artery or vein. In the third stage, we validated image annotations via network-level approaches. The final step is to disambiguate conflicting pixel labels between manual labelled mask and automatic predicted mask (generated by segmentation model B). In this workflow, single trained human annotator, who performs multi-stage annotation and subsequent label disambiguation, can substantially control the inter-annotator variability and intra-annotator variability.

### Image acquisition

The original colour fundus images are taken from task A of the IDRiD Challenge (held in *IEEE International Symposium on Biomedical Imaging* on April 4th, 2018)^24^. There are 54 images for training and 27 images for testing. We resized original images from 4288 × 2848 into 1024 × 1024 pixels with redundant image background (useless black pixels) cropped. Circular retinal field of view (FOV) is highlighted and the aspect ratio of FOV is kept unchanged. The story behind the selected 1024 is this number is a power of two^28^ occurring frequently in computer applications. Meanwhile, there is a great trade-off in terms of annotation time and the calibre of tiny vessels. It often takes more time to annotate tiny vessels with less than 3 pixels especially for an image with small FOV dimension. Our image down-sampling process aims to maintain small lesions like the microaneurysms still visible in a retinal image. In the original images of IDRiD dataset, any human labelled lesion whose area is less than 15 pixels will be disappeared from our down-sampled images. The missing percentages of the microaneurysms and hard exudates are 2 out of 3,497 and 18 out of 11,642 respectively. Contrast enhanced images could assist vessel boundary delineation and tiny vessel recognition during vessel labelling. In this work, the selected image enhancement methods are contrast-limited adaptive histogram equalization (CLAHE) and local contrast enhancement (LCE)^29,30^. The image transformation and enhancement algorithms are available in the released code package.

### Automated vessel segmentation

Pixel-level vessel labelling from scratch is an arduous task. Our approach is performing manual correction on a pre-segmented vessel image that is commonly adopted in community^31^. VGAN^32^ was a state-of-the-art DL model for retinal blood vessel segmentation some years ago but still performing excellently. One VGAN model trained on DRIVE database^33^ is used to detected vessel pixels. The input fundus image is resized to 640 × 640 and the output soft prediction (vessel probabilities ranged in [0,1]) is subsequently reshaped from 640 × 640 to 1024 × 1024 dimension. Binarized vessel images are extracted by Sauvola’s local adaptive thresholding method^34^ that can better preserve the connectivity of tiny vessels according to our experimental results. For each fundus image, we created a corresponding mat file (“mat” is the data container in MATLAB) putting the original retinal image, two enhanced images and segmented binary vessel together for the coming pixel-level vessel correction in CARL. The mat file creating code is also open-sourced. Our best practice of vessel annotation suggests the start-up vessel segmentation model had better be trained on another database for a higher segmentation quality. We believe other contemporary retinal vessel segmentation models^7–9^ instead of VGAN can also work well in practice.

### Vessel pixel correction

Misclassified pixels in binary vessel images can be categorized as **false-positive** pixels (background pixel wrongly classified as vessel pixel) and **false-negative** pixels (vessel pixel miscategorised as background pixel). We placed a binary vessel image over the fundus image with a changeable alpha parameter to control transparency of the vessel mask. The background fundus image is also switchable between the original and enhanced images. Our designed pixel manipulation tools can remove or add pixels from current vessel map. The vessel correction time is uncertain and it mostly depends on the accuracy of vessel pre-segmentation and technical skills of the human annotator. In this work, only one trained annotator was responsible for the correction work so as to prevent inter-annotator bias.

### Artery/Vein separation

**Fig. 3:**
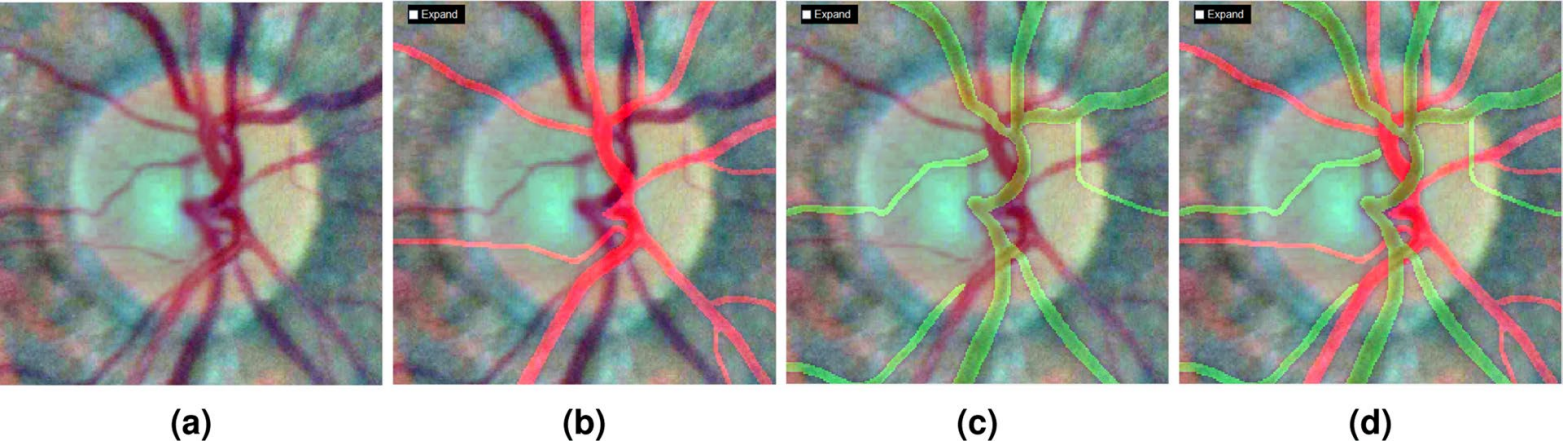
Artery/vein separation relating to the OD region. (**a**) Fundus image enhanced by LCE approach. From (**b**) to (**d**), the enhanced image is covered by artery mask, vein mask and A/V mask respectively (this view is the real annotation environment for human annotator in CARL software). Noticeably, we use green colour instead of blue to show venous pixels for a stronger background contrast. There are two visually isolated small vessels (difficult to locate the vascular root source from the given image) labelling as venules in (**c**). Their A/V labels could be predicted based on the topology feature.

A fact that calibre of veins are significantly wider than that in arteries^35^ indicates there could be more venous pixels than arterial ones in a retinal image. It is later verified by the statistical results of our annotated A/V masks (mean percentage of arterial and venous pixels are 41.91% and 58.09%). Therefore, to reduce the annotation time, our A/V labelling strategy is separating veins from binary vessel image, then masking out labelled veins from the original vessel image. At arteriovenous crossovers, manual image completion is used to link disconnected arterial vessel segments. Table 1 shows the A/V vessel characteristics in terms of colour, calibre, crossover, light reflex and topology. It is worth mentioning that we rigorously classified every vessel pixel into three classes (artery, vein or crossing). Our A/V masks are different from some existing datasets (*e.g*. RITE^36^, LES-AV^31^ and HRF-AV^30^). In their A/V masks, uncertain pixels are labelled and they mostly refer to intertwined large central A/V vessels inside the OD and disconnected small vessels outside the OD. Notwithstanding, A/V labels of these uncertain vessels could be recognized taking advantage of our specially designed CARL but requiring a little effort. We zoomed into the region of interest, switched to enhanced background image, located branching points of central A/V vessels and tracked A/V trees. One fundamental rule is that vascular loop is not allowed in the traced vascular tree. Figure 3 shows a zoomed OD region and disentangled A/V vessels. The A/V labels of isolated tiny vessels (Fig. 3(c)) can actually be determined by the crossover and topology features described in Table 1.

**Table 1:** Classification of retinal arteries and veins in 2-D fundus image based on five different characteristics.

Features	Differences between arteries and veins
Colour	Central veins have darker colour than central arteries. Not applicable to tiny vessels.
Calibre	Veins have wider diameters than adjacent arteries.
Crossover	Crossover can be categorised into artery over vein and vein over artery. AVN can only be seen when an arterial vessel is crossing over a venous one.
Light reflex	Veins show a smaller central light reflex.
Topology	Veins and arteries are commonly alternate to each other near the OD region.

The A/V annotation procedure in CARL software is as follows. We firstly chose “Vein Vessel” from “Vessel Types” popup menus. Colour fundus image will be overlaid with a binary vessel mask just like the exemplar in Fig. 3(b,d). We zoomed into a crossover region (typically connects 4 to 6 vessel segments), identified venous segments, disconnected and removed arterial segments. We named this A/V identification process as structure-level vessel annotation. Segments between two crossovers will share the same vessel type. It is also possible to categorise a crossover into **artery over vein** or **vein over artery**. Crossover type is crucial information for predicting vessel position from 2-D fundus images without the help of 3-D optical coherence tomography scans^37^. At this annotation stage, we paid special attention to vessel boundaries and crossovers where vessel edges are always hard to determine^38^.

### Vessel tree tracking

Extending from our previous work^10^, we constructed vascular graph based on the A/V mask and measured both the topological and geometrical properties of the vascular trees. The landmark points (bifurcation or terminal) are selected as vertexes in graph. The vertexes are interconnected by a set of edges (vessel skeleton segments). In this paper, **vessel skeleton segment** is an image patch contains a short segment extracted from vessel centreline image. It connects two anatomical landmark points in the vessel centreline image. And **vessel mask segment** indicates an image patch combining a vessel skeleton segment and its cross-sectional vessel pixels. It can be obtained by mapping the vessel skeleton segment to binary vessel mask. In this stage, we tracked all vascular trees, modified potential topological errors and further checked pixel annotations of vessel abnormalities.

#### Vessel skeletonization

**Fig. 4:**
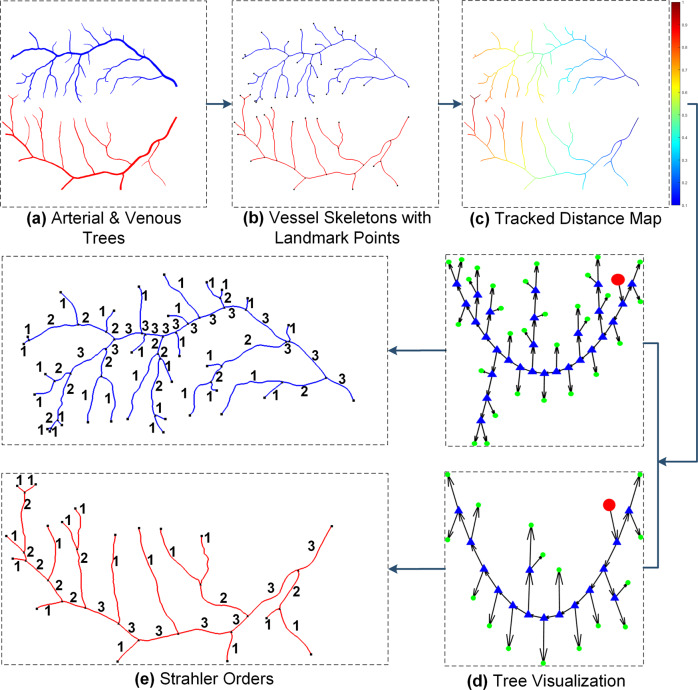
An example of vascular tree tracking. (**a**) Red and blue pixels indicate arterial and venous vessels. (**b**) Vessel skeleton image with landmark points in black (it is represented as a geometric tree or graph). (**c**) Normalized pixel distance away from the rightmost pixels. (**d**) Abstract directed graphs for arterial and venous vascular trees (red dots, blue triangles and green dots are starting, bifurcation and ending vertices). (**e**) Strahler orders assigned to vessel skeleton segments.

Vessel calibre varies from 1 to 20 pixels in RETA benchmark. Vessel skeletonization strives to reduce both thick and thin vessels to 1-pixel width representations. As one of tubular structures, retinal blood vessels can be presented as the envelope of a family of disks with continuously changing centre points and radii^39^. A desirable vessel skeleton image demands no spurs (spurious lines) on the extracted centrelines. In this study, we implemented Lee’s skeletonization method^40^ to extract vessel skeletons without any unwanted spur on vascular boundaries or endings. However, it failed to detect accurate vascular centrelines at FOV border where partial cross-section line of the vessel segment is outside the FOV. In this case, human intervention is required to correct these regions. We performed above approach on the arterial mask and venous mask separately. Figure 4(a) shows two vascular trees and the resultant vessel skeleton image is in Fig. 4(b).

#### Graph construction and visualization

We constructed both geometric and abstract vascular graphs. Vessel skeleton pixels of the OD region are removed. The geometric and topological features are usually impossible to estimate for the intertwined central vessels inside the OD. We selected three different types of points from the vessel skeleton image as vascular graph vertices. A 3 × 3 kernel filter is employed to calculate the number of neighbours for all pixels in the vessel skeleton image. Each skeleton pixel will have 1 to 4 neighbours. We defined the pixel that has at least three neighbours as bifurcation vertex. Starting and ending vertices are the skeleton pixels with exactly two neighbours. The starting vertex is generally close to the OD boundary. We removed the bifurcation, starting and ending pixels from the vessel skeleton image and obtained disconnected vessel skeleton segments. The next step is to build an adjacency matrix for all identified vertices. We proposed a graph region growing method to track vascular flow from a starting vertex. Vessel skeleton segment is used to connect two vertices. Vessel skeleton image with landmark points in Fig. 4(b) is represented as the geometric vascular graph. The abstract graph of every traced vascular tree is visualized by TreeVis tool^41^. Fig. 4(d) displays two vascular abstract graphs. Red and green dots indicate the starting and ending vertices.

#### Topological abnormalities detection

A normal rooted A/V vascular tree is a binary tree where a parent vertex only connects two child vertices without any graph circle. We proposed bifurcation vertex validation and graph circle detection methods to detect topological errors based on this assumption. For any bifurcation vertex, we computed the number of connected edges while assigning Strahler numbers to the graph edges (Fig. 4(e)). Strahler stream order is designed to reflect the morphology of vascular tree^27^. The human annotator was asked to validate abnormal bifurcation vertex with more than 3 connected edges. There is an unexpected case when 2 vessel bifurcations are extremely close in Euclidean space and the graph construction method inevitably merges them as 1 vertex. As for graph circle detection, we created a minimum spanning tree (MST) of each abstract vascular tree (AVT). Graph circle exists if there is any edge in AVT but not in MST.

#### Geometrical abnormalities detection

Three significant geometrical properties for retinal blood vessels are vessel calibre, bifurcation angle and tortuosity. Most vascular abnormalities are closely related to changes of these geometrical properties. Diameter changes are distinctly visible in venous beading, arteriolar/venules narrowing, branch retinal vein occlusion and AVN. Venous loop is one of tortuosity patterns^42^. We extracted the vessel mask segment for each vessel skeleton segment. Vessel diameter estimation method^43^ is applied to measuring vessel diameter of each centreline pixel from vessel mask segment. We tracked two child edges connecting to a bifurcation vertex and computed the bifurcation angle between them. In practice, bifurcation angle computation is restricted to a 10-pixel circular searching area of the vessel skeleton image. Vessel tortuosity index is also calculated for each vessel skeleton segment via Khansari’s approach^5^. We defined vessel geometrical abnormalities meeting any of the following condition: (1) focal calibre variation >3 pixels; (2) mean calibre of a parent vessel segment < mean calibre of any child vessel segment; (3) bifurcation angle >150°; (4) vessel tortuosity index >5. An experienced human annotator was assigned to review and update vessel annotations for all these candidate abnormalities. In this stage, we were capable of identifying different types of vascular abnormalities and producing thick/thin vessel masks based on the calculated geometrical features. The mean calibre of a thick vessel segment is assumed to be larger than 5 pixels in RETA. The rest of vessel segments are marked as thin. One thick/thin exemplar is shown in Fig. 1(g).

### Disambiguation of pixel labels

In the labelled vessel images, noisy pixels are more likely to be introduced at vessel boundaries and neglected by human annotators. Discovering and revising label noise could greatly improve vessel annotation quality. The noise identification method we would recommend is to train a vessel segmentor (another VGAN model) on the labelled vessel images and identify potential incorrect labels based on the predictions of this segmentor. This is a popular label cleaning approach based on the assumption that misclassified pixels can probably be noisy pixels^44^. We compared the predicted labels with training labels and categorized non-consensus pixels into two noise types, unlabelled and mislabelled. We utilised CARL software to inspect these noisy labels. A final version of vessel annotation for a retinal image is obtained after removing mislabelled pixels and joining unlabelled pixels. This operation essentially controls the intra-annotator variability.

## Data Records

Users can access RETA benchmark by visiting the public repository Figshare^45^ or our website at https://www.reta-benchmark.org. The shared data includes annotated binary vessel masks and different kinds of supporting materials. RETA comprises six compressed RAR files (each file contains a folder), namely “codes”, “images”, “mats”, “models”, “software” and “supports”. Vascular annotations are saved in the “images” folders. You might also feel interested in the raw annotation data that saved in the “mats” folder. The “supports” folder consists of various supplementary data to support this study. We will introduce the contents of “codes”, “models” and “software” folders in the Code Availability section.

In the “images” folder, we divided the images into “train” and “test” subfolders. What should be pointed out is that the training and test sets are already explicitly specified in the IDRiD Challenge. There are 54 and 27 images in the training set and the test set. In each subfolder, colour fundus images, fundus masks and vessel masks are stored in the “img”, “mask”, “vessel” folders respectively. Please be aware that colour fundus images are resized images not the original images from the IDRiD Challenge. A fundus mask, also known as the FOV mask, refers to a binary image indicating all captured retinal pixels by the fundus camera. It is reported that unbiased model performance had better be evaluated only in the FOV region^46^. The fundus and vessel masks are saved in PNG format (JPG format is a terrible option for saving binary images). They are titled as “*_mask.png” and “*_vessel.png” where “*” is the file name of corresponding colour fundus image. Vessel annotations of the test set are invisible to the public and it serves the purpose of model auditing^47^. **For more information about online model evaluation, please refer to our website**.

In the “mats” folder, we provided custom MATLAB mat files created in this work. Users could import these files into CARL and examine the tiny or complex vessel structures using its integrated toolbar. **Here, we sincerely call for independent observers’ feedback on any inappropriate vascular annotation that the contributors may mislabel**. All the mat files are named as “*_labeled.mat” and “*” indicates colour fundus image name. We stored all parameters relating to an image into a structure array named as “MAT”. Table 2 lists each structure field and its brief description. The height and width of an image should be divisible by 2 in order to meet the requirement of our image preprocessing algorithm. The changes of image height and width will be recorded in “dim_change” structure if the input image has an odd image height or/and width. The FOV region of a retinal image is cropped by a bounding box defined as [cropped_row, cropped_col, cropped_left, cropped_right]. The parameters are stored as four subfields of the “pos_data” structure. Pixel coordinate is written as (*y, x*) to present (*row, col*) in MATLAB image coordinate system.

**Table 2:** Data structure of custom mat file for CARL software.

Structure	Field	Description
MAT	I	A 3-D integer array denotes a colour fundus image
	I_cropped	A 3-D integer array denotes a fundus image cropped from I (see pos_data structure for cropping parameters).
	enhG_cropped	A 3-D integer array denotes the CLAHE enhanced image of I_cropped.
	enhC_cropped	A 3-D integer array denotes the LCE enhanced image of I_cropped.
	mask_white_o	A 2-D logical array denotes the fundus mask corresponding to I.
	mask_white	A 2-D logical array denotes the fundus mask corresponding to I_cropped.
	dim_change	A structure array contains the changing indicators of image dimension.
	pos_data	A structure array specifies image cropping parameters.
	annotations	A structure array comprises all kinds of pixel-level annotations.
dim_change	row	Boolean 1 and 0 denote that the height of an original fundus image is odd or even respectively. If 1, the first row of the image will be removed.
	col	Boolean 1 and 0 denote that the width of an original fundus image is odd or even respectively. If 1, the first column of the image will be removed.
pos_data	ori_row	An integer denotes the original image height of I.
	ori_col	An integer denotes the original image width of I.
	cropped_row	An integer represents the image height of I_cropped.
	cropped_col	An integer represents the image width of I_cropped.
	cropped_left	An integer array specifies upper-left location (*y, x*) of the cropping bounding box.
	cropped_right	An integer array specifies bottom-right location (*y, x*) of the cropping bounding box.
	extension	An integer array specifies the number of padded black pixels to the four boundaries of a fundus mask if its shape is not circular.
	row	An integer denotes the height of the original fundus image after dimension change.
	col	An integer denotes the width of the original fundus image after dimension change.
annotations	label	A string indicates the annotation type. In CARL, ‘BV’ indicates blood vessel.
	category	A numerical value for the given label. Category of the ‘BV’ is 4.
	sure_inds	Linear indices of all pixels belonging to the given category.
	unsure_inds	Linear indices of candidate pixels for the given category.

## Technical Validation

In the process of producing a high-quality binary vessel mask, we gained additional vessel annotations from different annotation stages. The extra annotations include A/V mask, skeletons, bifurcations, trees and abnormalities as shown in Fig. 1. The guaranteed quality of A/V annotation seems of greater importance because it could ensure reliable annotations of vessel skeletons, bifurcations and trees. Also, a binary vessel mask is actually the union of arterial and venous masks. What we have to point out is this paper’s workflow is quite different from the current situation of retinal image annotation in community. The A/V labels of RITE and HRF-AV datasets are identified and produced from established DRIVE and HRF vessel databases. However, in RETA, we obtained binary vessel masks after strict quality control of their A/V labels. To the extent of our knowledge, vessel labeling guidelines and quality evaluation protocols are still lacked in literature. We wish this paper could raise awareness of related topics. In this section, we dedicated to validating the reliability of our final binary vessel masks by means of subjective and objective approaches.

The most common method for evaluating quality of image segmentation is subjective evaluation. Nevertheless, it can be highly tedious and time-consuming for human graders. In retinal image analysis field, a well studied topic is quality grading of retinal images^48^. Visibility of retinal vessels is an essential and important feature for both manual and automated quality grading systems. To objectively evaluate segmentation quality, unsupervised quantitative metrics such as object shape are designed for the segmented objects^49^. In most cases, appropriate measures are hard to define especially for the complicated retinal vascular structures. Galdran *et al*.^50^ proposed a similar regression model learning to recognise the differences between computer-degraded and human-labelled vessel masks. This model devotes to predicting quality score of any input vessel segmentation image. From our perspective, disadvantages of this model include (1) inability to judge potential false-positive and false-negative vessels from merely a segmented vessel image; (2) model is prone to be biased using rule-based degraded artificial vessel images as its training set. In a word, designing an advanced objective quality evaluation metric is a grand challenge.

### Subjective quality evaluation

**Fig. 5:**
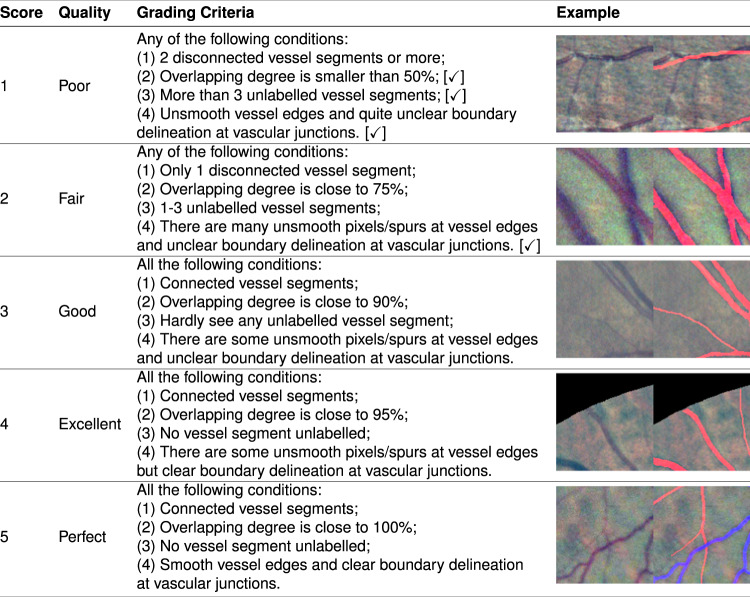
Subjective grading scale for vessel annotation quality assessment. Overlapping degree % is subjectively predicted by human grader. A pair of image is provided for each quality level. The left image is a 128 × 128 LCE enhanced image patch and the right one shows labelled vessel pixels. For the “Poor” and “Fair” quality levels, a [✓] logo will be attached if corresponding condition is met.

We proposed four heuristic metrics for subjective vessel quality evaluation. They are segment connectivity, overlapping degree, mislabelled quantity and edge smoothness. Segment connectivity demands the pixels of a vessel segment must be connected and not broken. Overlapping degree measures the overlap between marked and ground-truth blood vessel pixels. It is a region based metric similar to Dice coefficient. Mislabelled quantity refers to the number of unlabelled ground-truth and labelled artificial vessel segments. Edge smoothness is established on the assumption that normal blood vessel has smooth boundaries like a tube. The jagged vessel edges are caused by inexperienced image annotators. We devised a 5-level grading scale for the subjective evaluation of labelled vessel images. Figure 5 displays five quality classifications with typical images. Generally speaking, “Poor”, “Fair” and “Good” quality levels with explicit grading criteria are adequate for vessel annotation quality assessment. The design of higher quality levels (“Excellent” and “Perfect”) is used to reward vessel annotation with smooth and precise vascular delineation particularly at the vessel boundaries and junctions.

We studied the vessel annotation quality of RETA and 11 open datasets (listed in chronological order, STARE^51^, DRIVE^33^, ARIA^52^, CHASE_DB1^53^, HRF^54^, DRHAGIS^55^, UoA-DR^56^, LES-AV^31^, DualModal2019^57^, AFIO^58^ and ORVS^59^). Image dimension of different dataset is various (the smallest dimension is 565 × 584 pixels from DRIVE and the largest dimension is 4752 × 3168 pixels from DRHAGIS). To simplify and standardise the subjective evaluation process, we randomly selected only 5 images from each image set and created mat files using our image transformation approach. Vessel annotations of the FOV region are rescaled to 1024 × 1024 pixels. The resized vessel images show identical vascular structures to the original ones with the exception of some large-dimension images in HRF and DRHAGIS. Tiny vessels with widths between 1 to 3 pixels will become disconnected in our transformed images. Please be alert to the grading results of HRF and DRHAGIS that might be adversely affected by disconnected vessels.

One trained human grader evaluated the quality of labelled vessel masks with the aid of annotation quality rater (AQR) tool embedded in CARL. AQR will divide a 2-D image of 1024 × 1024 pixels into image patches of 128 × 128 pixels. Grading process is single-blind and the grader do not know the source of images. The human grader gave a score (0–5) to each patch during the grading process. Score 0 denotes no visible vessel in the present image patch. We also visualized the quality scores by means of covering a colour checkerboard over the vessel image (available in the “supports” folder). Statistical results (mean ± 2 × standard deviation) of each database are presented in Table 3. All image patches of score 0 are excluded.

**Table 3:** Subjective quality assessment results of vessel annotations on 11 public datasets and RETA.

Rank	Dataset	Quality Score
1	RETA	3.0880 ± 1.4306
2	DualModal2019	2.4021 ± 1.5130
3	DRHAGIS	2.2792 ± 1.4632
4	LES-AV	2.2625 ± 1.6988
5	CHASE_DB1	2.2035 ± 1.2230
6	HRF	2.1925 ± 2.2646
7	AFIO	2.1715 ± 0.9754
8	ORVS	1.9699 ± 1.6102
9	ARIA	1.7402 ± 1.0803
10	STARE	1.6745 ± 1.1082
11	DRIVE	1.6630 ± 1.2630
12	UoA-DR	1.1187 ± 0.6480

RETA dataset ranks on the top of Table 3 and achieves the biggest mean score. The grading scores of well-known STARE and DRIVE are not high. In our opinion, one possible reason is limited hardware and software facilities for retinal vessel annotation task in the early period of 21st century. Moreover, the FOV dimensions of STARE, DRIVE and ARIA are approximately 647 × 647, 535 × 535 and 750 × 750 pixels. Small FOV dimension actually makes vessel annotation task troublesome since there are many 1-pixel width tiny vessels. The surprising high standard deviation of HRF is caused by disconnected capillaries in the resized 1024 × 1024 images. This does not mirror the actual quality of vessel annotations. So is the DRHAGIS database. We manually removed outlier image patches (score = 1) with visible broken vessels and obtained revised scores of 2.7066 ± 1.9551 and 2.3309 ± 1.3970 for HRF and DRHAGIS respectively. From the subjective evaluation results, our RETA is a high quality vessel dataset until now. The success of applicable vessel segmentation models depends on high-quality labelled training data. Model validation and auditing also benefits from a reliable test set. We believe RETA will enable fair comparisons among different approaches.

### Objective quality validation

In this section, we introduced four validation methods to objectively evaluate the annotation quality of vessel masks. Hole detection and connected component analysis are two basic image processing techniques. Human observers are in the loop of validation process to classify a detected region into noise or vessel. Furthermore, we exploited fractal dimension (FD) of the retinal vasculature as a clinical metric for evaluating the annotated vessel images since the change of FD is associated with disease progression^60^. Finally, we evaluated the cross-dataset vessel segmentation performance of 12 segmentation models trained on different vessel database. It is well known that training set related factors influencing the model generalization ability are the scale of training set and the amount of label errors. We used the same architecture of vessel segmentation model and controlled the training strategy, learning epochs and image scale identical. Then, the impact of noisy labels on the model generalizability can be objectively and fairly observed.

#### Hole detection

A hole is a set of black pixels concerning a white object in a binary image. The existence of holes inside a vessel segment will lead to inaccurate vascular topology. Also, its skeletonization result tends to be erroneous. We used hole detection algorithm to discover potential holes in the labelled vessel masks. A flood-fill operation was applied to arterial mask and venous mask separately. There is no hole belonging to false-negative pixels among the detected candidate holes. In total, 6 holes were verified as venous loop in 6 images (“IDRiD_40.jpg”, “IDRiD_68.jpg”, “IDRiD_69.jpg”, “IDRiD_74.jpg”, “IDRiD_76.jpg” and “IDRiD_77.jpg”). Only 2 holes are found in the OD region from two images (“IDRiD_46.jpg” and “IDRiD_72.jpg”). We call them as “Loops in OD”. As mentioned in the Artery/Vein Separation section, loops are unacceptable vascular patterns for a normal vascular tree. However, we have to retain the “Loops in OD” caused by intertwined central vessels of the OD region.

#### Connected component analysis

Isolated small area or connected components (CC) is a particular region of interest in the validation stage. A small CC might be false-positive vessel pixels or a pixel block disconnected from a large CC. Human annotators are apt to ignore these small areas during the process of labelling. Technical validation of these regions could identify the wrong annotation and improve annotation quality. Connected component analysis is employed to locate candidate CCs. We used this approach to analyse both the binary arterial and venous masks. Any CC whose area is smaller than 100 pixels will be double-checked. We identified 36 small CCs (11 CCs belong to arteries and 25 CCs are part of veins) and none of them was classified as false-positive.

#### Fractal dimension analysis

Fractal dimension analysis is a mathematical approach to measure the geometric complexity of retinal vascular trees. We used box-counting dimension^61^ to calculate the FD of manual-labelled vessel images. Two trained human readers graded the severity levels of DR and diabetic macular edema (DME) for all 81 colour fundus images in RETA according to the International Clinical Diabetic Retinopathy Scale^62^. Measuring areas of FD include the entire FOV region and standardized macula region (radius of the macula is 0.6 times the distance of macula center to OD center). DME is known to be closely related to vascular density changes within the macula region.

**Fig. 6:**
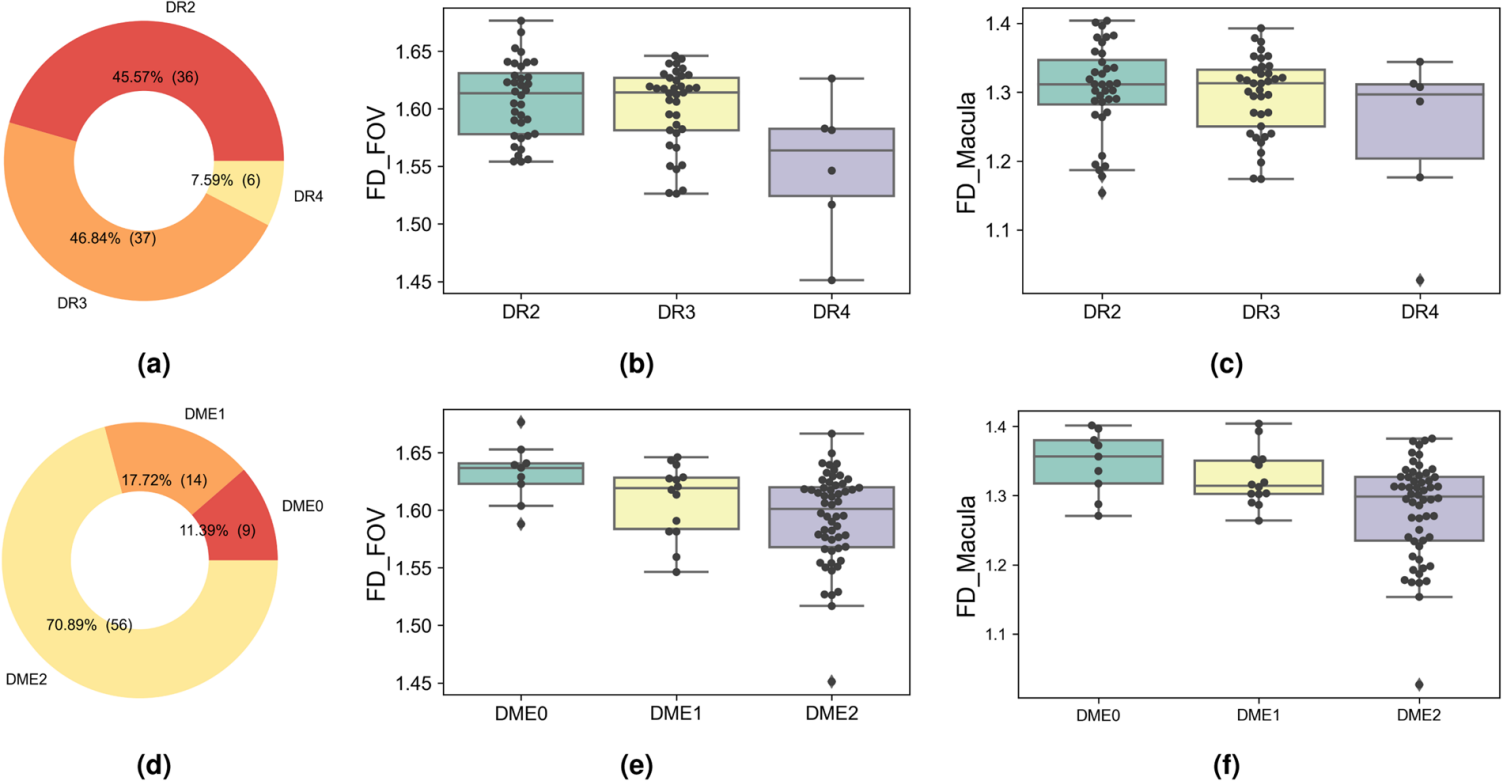
Doughnut charts of study group distribution and box plots of measured FD values. (**a**) and (**d**) show image distribution for DR and DME respectively. (**b**) and (**e**) are box plots of the FOV region. (**c**) and (**f**) exhibit box plots of the macula region.

Doughnut charts in Figure 6 are image distribution of different DR and DME groups. Two images (“IDRiD_04.jpg” and “IDRiD_45.jpg”) are excluded from this study since they are OD-centered fundus images. DR2, DR3 and DR4 refer to moderate non-proliferative, severe non-proliferative DR and proliferative DR. DME0, DME1 and DME2 indicate normal, non-clinically significant DME and clinically significant DME. The box plots in Fig. 6 showed the mean FD and 95% confidence intervals. We only compared FD values of the DR2 and DR3 groups since the sample size of DR4 group was not big enough. T-test showed there was no significant difference between them for the entire FOV (*p* = 0.3892) and macula region (*p* = 0.5520). Meantime, the FD of DME0 and DME2 was significant (*p* < 0.05) in both regions but the FD of DME1 and DME2 was only significant (*p* < 0.05) concerning to the macula region. The progression of DME severity was found to be associated with lower FD. This finding was consistent with previous clinical studies^60^.

#### Cross-dataset benchmark

We investigated the impact of noisy labels on cross-dataset generalizability in a controlled experiment. Cross-dataset refers to training the segmentation model in one dataset and testing it in another. Noise labels of the training set are assumed to have negative effects on cross-dataset generalization. To control for the effect of various factors, such as model architecture, training strategy and the size of the training set, we used VGAN as the baseline segmentation model and built a training set of the same image dimension and a total number of images for each dataset.

Table 4 shows the split strategy of training and test sets for 12 datasets. The training and test sets are already specified in DRIVE, DualModal2019, ORVS and RETA. For HRF, we adopted Yan’s dataset dividing approach^63^. LES-AV, CHASE_DB1, ARIA, DRIVE and UoA-DR are equally divided into training and test sets. We only selected vessel images labelled by the 1st observer in DRIVE and CHASE_DB1. For each database, the colour fundus images, FOV masks and vessel masks are transformed into 1024 × 1024 pixels. The newly constructed data is available in the “supports” folder.

**Table 4:** Dataset configuration for cross-dataset evaluation.

Dataset	Rotation angle(s)	Total image	Training/test set specification
RETA	24	81	There are 54 images for training and 27 images for testing.
DualModal2019	10	30	There are 24 images for training and 6 images for testing.
DRHAGIS	9	40	The first 5 images of each subgroup (glaucoma, hypertension, DR and age-related macular degeneration) are in the training set (20 images) and the remaining images are in the test set (20 images).
LES-AV	6, 25	22	The first 11 images are in the training set and the rest 11 images are in test set.
CHASE_DB1	6	28	The first 14 images are in the training set and the rest 14 images are in test set.
HRF	6	45	The first 5 images of each subset (healthy, DR and glaucomatous) are for training (15 images in total) and the remaining 30 images are for testing.
AFIO	23	100	The first 50 images as the training set and the rest 50 images as the test set.
ORVS	18	49	There are 41 images for training and 8 images for testing.
ARIA	30	143	The first 70 images are in the training set and the rest 73 images are in test set.
STARE	9	37	There are 20 images for training and 17 images for testing.
DRIVE	9	40	There are 20 images for training and 20 images for testing.
UoA-DR	45	200	The training set comprises the first 28 images of health group, the first 57 images of non-proliferative DR and the first 15 images of proliferative DR. The remaining 100 images are in the test set.

We utilised image rotation and flipping to enlarge the training set of each dataset. The rotation angle of each dataset is listed in Table 4 and our data augmentation code is also available in the “supports” folder. Each training set consists of 1,600 images after data augmentation (may require manual deletion of extra images). We trained a VGAN model on each training set. The model input is a 1024 × 1024 colour retinal image and output is single channel vessel prediction of the same image dimension. Optimizer is Adam and initial learning rate is set to 2e-4. We did not change the learning rate during model training. The loss function is binary crossentropy and batch size is 1. The training strategy of VGAN is identical for all datasets. VGAN comprises a generator network and a discriminator network. The generator network is trained for 1 epoch before iteratively training the discriminator and generator networks. The number of training rounds is set to 6. In each round, the discriminator and generator take turns to train for just 1 epoch. We loaded the last saved model weights and calculated vessel segmentation performance within the FOV region of resized vessel images from the test set.

**Fig. 7:**
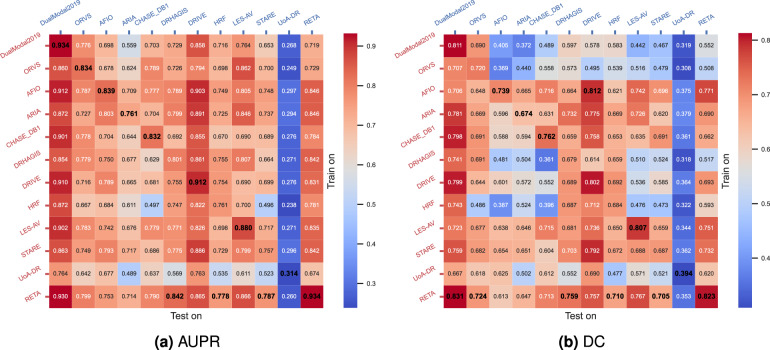
Benchmarking the retinal vessel segmentation performance of 12 vessel datasets in a controlled experiment. Each row of (**a**) AUPR or (**b**) DC indicates the measured performance for each model trained on the source dataset. The best segmentation performance for each test set (the column of matrix) is in bold.

Figure 7 shows cross-dataset evaluation results in terms of the area under precision recall curve (AUPR) and Dice coefficient (DC). The best testing performance of each column is in bold. Elements of the main diagonal indicate model performance achieved from training and testing on the same dataset. In general, the values of the main diagonal should be the largest due to the smallest domain gap between two subsets from the same database. Many interesting findings could be made from this benchmarking results. First and foremost, the model trained on RETA obtains 4 and 6 top results in Fig. 7(a,b) respectively. That demonstrates RETA model has the best cross-dataset generalizability. For the model trained on UoA-DR dataset, both the AUPR and DC metrics indicate inferior segmentation performance. It supports the subjective assessment results as illustrated in Table 3. We believe the model generalizability is highly associated with noisy labels of the training set. But we are unable to estimate the amount of noise in each dataset solely based on this benchmark study. We also observe that the model trained on HRF performs not well on CHASE_DB1 and STARE test sets, and the domain gap probably causes it. From the results of DC metric, models trained on AFIO, ARIA, CHASE_DB1, LES-AV, STARE and RETA perform better. From our point of view, it seems not meaningful to evaluate vessel segmentation performance on the test set of UoA-DR dataset because its vessel annotation quality is unacceptable. Finally, what we have to clarify is the performance of HRF model might be underestimated owing to degraded thin vessels of our resized images.

## Usage Notes

The RETA benchmark is licensed under the Creative Commons Attribution 4.0 International License. A proper citation of this paper is expected if you use vessel annotations or any supplementary data from RETA in your work. We wish to discuss three issues may be essential for readers or beginners who are going to research this topic.

### Pay attention to noisy labels of the dataset before developing your segmentation models

Noisy labels are more often in medical domain due to costly annotation and label variability. Training with noisy labels is an active research topic^44,64^ in image segmentation task. It would be exciting to see a segmentation model could achieve a strong generalization capability learning from noisy labels. We strongly encourage users to develop new techniques using previous open vessel database and evaluate vessel segmentation performance on the test set of RETA. Users can also validate existing robust training approaches on RETA. And evaluate their vessel segmentation performance on the test set of RETA. Besides, one important issue but easily ignored^32,63^ is the noise can also be accidentally generated at data augmentation stage for retinal vessel images. We have recognized the presence of disconnected tiny vessels (less than 3 pixels in calibre) in the rotated images and its negative influence on model performance (*e.g*. the trained HRF models). Therefore, we discourage users to downsample the provided 1024 × 1024 binary vessel masks using a scale factor of $\frac{1}{3}$ or smaller. It will narrow the calibre of annotated vessels and the topological properties of tiny vessels may not be well preserved. Conversely, when upsampling a binary vessel image to a larger image dimension, anti-aliasing technique is recommended to be applied in order to get smooth vessel edges. Noise pixels can be easily introduced at jagged vessel edges.

### Selecting appropriate evaluation metrics for vessel segmentation task is nontrivial

A golden combination set {accuracy, sensitivity and specificity} is frequently adopted to indicate vessel segmentation performance in literature^9,46^. Other pixel-wise matching based metrics (*e.g*. area under the receiver operating characteristic curve) are also reported in previous papers. These metrics only present limited fairness for vessel segmentation evaluation^14,21^. One well known reason is the annotator variability problem. Varied vessel masks (both in vessel thickness and location) are labelled by 1st and 2nd observers in the DRIVE and CHASE_DB1 datasets. Furthermore, these metrics share a major drawback in that they overlook the important geometric and topological information of vascular structures. Quantitative measurement of some vascular biomarkers (*e.g*. FD) tremendously depends on accurate vascular topology. In addition, a fair metric should take the imbalanced pixel ratio (thick versus thin and vessel versus background) of retinal images into consideration. We suppose DC (also known as F-1 score) and AUPR are better options. DC numerically equals to F-1 score in the binary vessel segmentation task. Recently, advanced metrics like skeletal similarity^21^ and topological similarity index^65^ insensitive to vessel thickness might be alternatives for segmentation evaluation. For a more difficult assignment of large-scale model evaluation, we prefer a no-reference metric like the similar regression model^50^ since it seems impossible to annotate massive accurate vessel images (at least tens of thousands of images in the test set). The latest TREND^66^ and FIVES^67^ image sets only contain 82 and 800 vessel images. As a matter of fact, selecting unbiased and proper evaluation metrics is still an open resolved issue for the community members.

### Giving up developing basic image processing algorithms is never a good thing in the deep learning era

**Fig. 8:**
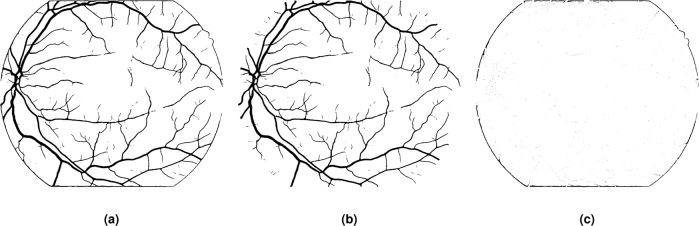
Effectiveness of border extension technique for artefacts removal. (**a**) and (**b**) are binary vessel images predicted from the original and preprocessed images. (**c**) is the difference image between (**a**) and (**b**).

To our understanding, DL technique is a fabulous tool to process images but it still shows limitations. One example we would love to share is the false-positive vessels detected at FOV border (Figure 8(a)). It might be caused by strong contrast since pixel intensity outside FOV is close to zero. And it can also be observed if you use z-score normalization method (that is, the mean of all pixels is 0 and the standard deviation is 1) as the image normalization method for DL model training and testing. A really simple image processing technique can solve this issue. We implemented an artificial pixel padding approach at the FOV border inspired by Soares’s preprocessing algorithm^68^. A MATLAB implementation named as “FOV_border_extension.m” is provided in the “codes” folder. Fig. 8(b) shows vessel segmentation result of the preprocessed image. To find black pixels in Fig. 8(a) that are not in Fig. 8(b), we get the border artefacts shown as in Fig. 8(c). False-positive vessels are effectively removed. This example demonstrates even DL-based blood vessel segmentation models can be easily fooled and further study of advanced DL models or other cutting-edge techniques seem necessary for researchers in both medical image analysis and computer vision domain.

## Data Availability

We would like to release the following software, algorithms and trained vessel segmentation model that used in the construction process of RETA benchmark. They can be found from the “codes”, “models”, “software” and “supports” folders. • **CARL software**. It could help users to investigate annotation details of vascular structures in a zoomed and magnified view. Users are also able to visualize and evaluate segmentation results of retinal anatomical structures and lesions using CARL software. It can work as a model auditing tool and is available in the “software” folder. • **Image transformation and enhancement**. The “codes” folder contains essential MATLAB source code of our image preprocessing algorithm. Users can use “IDRiD_cropImage.m” to crop original colour images of the IDRiD challenge. “IDRiD_restoreImage.m” is used to upsample labelled vessel masks. A script file named as “CARL_image2mat.m” can transform users’ private images into our custom mat files. • **Vessel segmentation model**. A pretrained vessel segmentation model and corresponding model inference code are in the “models” folder. Please read usage documentation before executing model inference. It may take some time to set up the running environment. • **Vessel centreline extraction**. We borrowed a Python implementation of the skeletonization method from “scikit-image” Python package to extract vessel centrelines. The parameter “method” of the used skeletonize function is “lee” in our experiment. • **Data augmentation**. We utilised basic image rotation and flipping to create a large-scale image set for model training. A multi-thread image augmentation code implemented in Python programming language is saved in the “supports” folder.
